# Feasibility of routine data collection on icu performance and activity in resource limited settings

**DOI:** 10.1186/2197-425X-3-S1-A162

**Published:** 2015-10-01

**Authors:** SMM Ali, MI Memon, V Ioos, TMU Pasha, S Afghan, F Tahir, A Zeb

**Affiliations:** Shaheed Zulfiqar Ali Bhutto Medical University Hospital, Islamabad, Pakistan; Service de Réanimation, Hôpital Delafontaine, Saint Denis, France

## Introduction

Routine collection and analysis of data allows critical care department to highlight the outcomes of past interventions and to identify the grounds for future improvement. Data on characteristic and outcomes of ICU patients are lacking in Pakistan. We report data collected in 2014 in a 9-bedded medical ICU of Shaheed Zulfiqar Ali Bhutto medical university hospital, Islamabad.

## Objectives

To determine the standardized mortality ratio (SMR), mean duration of ICU stay and mechanical ventilation and ICU acquired infection incidence rate during the year 2014 and to compare it with 2008 data.

## Methods

Since 2008, performance and activity data of the medical ICU are entered daily. A software (ICU e-monitoring^®^) was designed to enter patient demographic data, SAPS3 on admission and episodes of hospital acquired infections (ventilator associated pneumonia (VAP), catheter related blood stream infection (CRBSI)). Data were collected in 2014 with the same methodology as the 2008, previously published dataset ([1]) that served for comparison.

## Results

A total of 196 admissions recorded during the year 2014 (354 in 2008). 47.2% were males and 52.8% were females. Mean age was 32.1 years ± 15.3 SD (37.7 ± 18.9 SD in 2008).Mean SAPS3 score was 68 (44 in 2008). A total of 33% deaths recorded (47% in 2008). SMR was 0.71(1.09 in 2008). Mean duration of ICU stay was15.9 days ± 12.9 SD (9.3 days ± 8.9 in 2008) and mean duration of mechanical ventilation was 12.04 days (8.7 in 2008). Overall VAP rate was 42.3 cases/1000 ventilator days (16.7 in 2008). Rate of CRBSI was 17.2 cases/1000 CVC days (12.1 in 2013).

## Conclusions

It is possible to collect meaningful data on ICU performance and activity in resource limited settings, which are useful as internal and external comparator. There were major changes in our patient population characteristics between 2008 and 2013: number of patients decreased by 44%, SMR decreased from 1.09 to 0.71 and incidence rate of VAP and CRBSI increased by 153% and 42% respectively. Since 2008, intensive care medicine post-graduate training program was started in our institution and ICU admission criteria were defined and implemented. Longer duration of stay in ICU and mechanical ventilation, as well as variations in the case-mix of our patient population and improved clinical management may explain the observed changes. The high rate of device associated infections, as compared to published figures ([2]) requires urgent targeted interventions.

## Grant Acknowledgment

The ICU e-monitoring^®^software was funded through a grant from French Embassy in Pakistan.
